# Self-delivering prodrug-nanoassemblies fabricated by disulfide bond bridged oleate prodrug of docetaxel for breast cancer therapy

**DOI:** 10.1080/10717544.2017.1381201

**Published:** 2017-09-26

**Authors:** Shenwu Zhang, Jibin Guan, Mengchi Sun, Dong Zhang, Haotian Zhang, Bingjun Sun, Weiling Guo, Bin Lin, Yongjun Wang, Zhonggui He, Cong Luo, Jin Sun

**Affiliations:** a Department of Pharmaceutics, Wuya College of Innovation, Shenyang Pharmaceutical University, Shenyang, P. R. China;; b School of Life Science and Biopharmaceutics, Shenyang Pharmaceutical University, Shenyang, China;; c Key Laboratory of Structure-Based Drug Design and Discovery, Shenyang Pharmaceutical University, Ministry of Education, Shenyang, China

**Keywords:** Breast cancer, docetaxel, oleate prodrug, self-assembly, self-delivery

## Abstract

Breast cancer leads to high mortality of women in the world. Docetaxel (DTX) has been widely applied as one of the first-line chemotherapeutic drugs for breast cancer therapy. However, the clinical outcome of DTX is far from satisfaction due to its poor drug delivery efficiency. Herein, a novel disulfide bond bridged oleate prodrug of DTX was designed and synthesized to construct self-delivering prodrug-based nanosystem for improved anticancer efficacy of DTX. The uniquely engineered prodrug-nanoassemblies showed redox-responsive drug release, increased cellular uptake and comparable cytotoxicity against 4T1 breast cancer cells when compared with free DTX. *In vivo*, oleate prodrug-based nanoparticles (NPs) demonstrated significantly prolonged systemic circulation and increased accumulation in tumor site. As a result, prodrug NPs produced a notable antitumor activity in 4T1 breast cancer xenograft in BALB/c mice. This prodrug-based self-assembly and self-delivery strategy could be utilized to improve the delivery efficiency of DTX for breast cancer treatment.

## Introduction

Breast cancer is one of the most serious malignant tumors threatening to the female health all over the world (Siegel et al., 2016). In clinical practice, breast cancer is still largely incurable with high relapse rate (Bianchini et al., 2016). Chemotherapy represents one of the most common treatment strategies, playing crucial roles in breast cancer therapy. However, the chemotherapeutic efficacy is very limited and far from satisfaction. As the limited efficacy of current chemotherapy against breast cancer is a result of inefficient drug delivery, due to the off-target effects of most existing chemotherapeutic formulations (Bianchini et al., 2016).

Docetaxel (DTX), a typical antimitotic agent, is an important member of the taxane family and shows more potent antitumor activity than paclitaxel (PTX) (Baloglu &Kingston, 1999; Chu et al., 2016). DTX is mainly used for advanced breast cancer treatment in clinics (Liu et al., 2016; Ren et al., 2016b; Sorolla et al., 2016; Xu et al., 2016; Tan et al., 2017a; Wang et al., 2017). However, despite its excellent *in vitro* antitumor activity, the therapeutic efficacy of DTX injection (Taxotere) is greatly limited by poor drug delivery efficiency and serious excipient-associated toxicity (de Weger et al., 2014; Wu & Zheng, 2016). With the rapid development of nanoscience and technology, nanoparticulate drug delivery systems (nano-DDS) have been widely used in healthcare applications, especially for the high-efficient drug delivery of chemotherapeutic agents (Baetke et al., 2015; Luo et al., 2016; Wais et al., 2016; Yuan et al., 2016; Tan et al., 2017b). Nano-DDS can be accumulated in tumor site due to the well-reported enhanced permeability and retention (EPR) effect (Baetke et al., 2015; Wais et al., 2016). Despite all this, very few of the nano-formulations could be finally applied to the clinical treatment of cancer (Luo et al., 2012). Because the conventional nanomedicine (e.g. liposomes, nanoparticles (NPs) and micelles) exist obvious shortage in terms of preparation, drug loading efficiency, stability, drug release and even therapeutic efficacy (Luo et al., 2014), and there is still a great gap between the preclinical studies and the actual clinical application (Petersen et al., 2016). Therefore, new and more efficient drug delivery strategy should be developed to facilitate the clinical chemotherapeutic efficacy of anticancer drugs by further improving drug delivery efficiency.

In recent years, prodrug-based nano-DDS has attracted widespread attention (Luo et al., 2014). Prodrug-nanosystems, as novel self-delivering nano-platform assembled from anticancer drug conjugates themselves, have demonstrated their distinct drug delivery advantages in terms of drug-loading capacity, controlled and selective drug release and favorable antitumor efficacy (Luo et al., 2014). Our group has designed and fabricated a series of prodrug-nanoassemblies and the self-assembly mechanism and redox responsive drug release of prodrug NPs were investigated (Wang et al., 2014; Han et al., 2016; Luo et al., 2016). It turned out that multiple mechanisms involved in the self-assembly process of prodrug NPs, including disulfide bond insertion, intermolecular π – π stacking and structural flexibility of prodrugs (Wang et al., 2014; Luo et al., 2016). In addition, we found that disulfide bond bridge not only played important roles in self-assembly but also could facilitate more rapid and selective drug release in tumor cells when compared with other redox responsive linkages (Wang et al., 2014).

DTX is mainly used in the treatment of breast cancer in clinics, but its therapeutic effect is greatly limited by the inefficient drug delivery. To address the challenges of DTX drug delivery, in the present study, we intended to design disulfide bond bridged oleate prodrug of DTX and construct prodrug NPs based on the conjugate for breast cancer therapy. Firstly, a novel disulfide bond bridged oleate prodrug of DTX (DTX-S-S-OA) was synthesized and DTX-S-S-OA could self-assemble into prodrug NPs. Then, the antitumor efficacy of DTX-S-S-OA prodrug NPs was evaluated in 4T1 breast cancer cells *in vitro* and 4T1 breast cancer xenograft of BALB/c mice *in vivo*. To our knowledge, this is the first attempt to synthesize DTX-S-S-OA conjugate and to evaluate the antitumor efficacy of DTX-S-S-OA prodrug-nanoplatform against its major indication (breast cancer) *in vitro* and *in vivo*. Additionally, the endocytosis mechanism of prodrug-nanoassemblies was investigated in the present study for the first time.

## Materials and methods

### Materials

DTX was obtained from Dalian Meilun Biotech Co., Ltd, China. Oleic acid, N’-dicyclohexylcarbodiimide (DCC), 4-dimethylaminopyridine (DMAP), phenylmethanesulfonyl fluoride (PMSF), N-ethylmaleimide (NEM), DTT and acetic anhydride were bought from Aladdin Industrial Corporation, Shanghai, China. Dithiodiglycolic acid was purchased from Alfa Aesar (China) Chemicals Co., Ltd. Cell culture reagents were purchased from GIBCO, Invitrogen Corp. (Carlsbad, CA). MTT, trypsin-EDTA and coumarin-6 were purchased from Sigma-Aldrich, St. Louis, MO. DAPI was obtained from Vector Laboratories, Burlingame, CA. DiR was purchased from ATT Bioquest, Beijing, China. 1, 2-distearoyl-sn-glycero-3-phosphoethanolamine-N-[methoxy(polyethyleneglycol)-2000] (DSPE-PEG_2K_) was purchased from Shanghai Advanced Vehicle Technology L.T.D. Co. All other reagents and solvents mentioned in this article were of analytical grade. DTX-OA was synthesized according to our previous method (Sun et al., 2016a).

### Design and synthesis of DTX-S-S-OA

Oleic acid (10.16 mmol) dissolved in 4 ml of methylbenzene was added to P-toluenesulfonic acid (1.01 mmol) in 32.8 ml ethylene glycol (0.14 mol). The reaction was sustained with stirring for two hours under nitrogen protection at 110 °C. Thin layer chromatography (TLC) was used to monitor if the oleic acid reacted completely. The product was extracted with methylbenzene and was washed with the saturated NaHCO_3_ solution. Anhydrous sodium sulfate was used to remove the remaining water and then the product was filtered under vacuum condition. Finally, a transparent oily liquid was separated and obtained with column chromatography (*n*-hexane/ethyl acetate (30:1)) and the yield of oleic acid 2-hydroxyethyl ester was 56.4%.

2,2′-di-thiodiglycolic acid anhydride(1.25 mmol), EDCI (1.25 mmol and HOBt (1.25 mmol) were dissolved in 10 ml dichloromethane and then oleic acid 2-hydroxyethyl ester (0.83 mmol) dissolved in 1.5 ml dichloromethane was slowly added to the solution with stirring for 0.5 h under ice bath. The reaction temperature was transferred to 25 °C and stirring was continued for 12 hunder nitrogen. The reaction process was monitored by TLC. After the reaction, the solution was acidized with hydrochloric acid solution. The product was extracted with dichloromethane and the saturated NaCl solution was utilized to wash the organic phase layer. After washing, the solution was dried with anhydrous sodium sulfate, filtrated and evaporated. The objective product was depurated further with the column chromatography and a wine-colored oily liquid (2′-O-(2-oxo-2-(2-((Z)-oleoyloxy) ethoxy) ethyldisulfide) aceticacid) was obtained with a yield of 57.4%.

DTX (0.50 mmol), EDCI (0.52 mmol) and HOBt (0.52 mmol) was dissolved in 10 ml dichloromethane, the 2′-O-(2-oxo-2-(2-((Z)-oleoyloxy) ethoxy) ethyldisulfide) acetic acid in 2 ml dichloromethane was added to the solution with stirring for 0.5 h at 0 °C and 12 h at 25 °C under nitrogen. The post processing of the destination product was similar to the first two steps, which was washed, dried, filtrated, evaporated and then purified by the preparative liquid chromatography. Eventually, the destination product, a faint yellow solid (DTX-S-S-OA) was obtained and the yield was 49.5%.

### Preparation and characterization of prodrug nanoassemblies

Prodrug nanoassemblies were prepared by the nano-precipitation method according to our previous work (Wang et al., 2014). Briefly, 4 mg of prodrugs (DTX-OA or DTX-S-S-OA) and 1 mg of DSPE_2K_ (DSPE_2K_/DTX-OA =9.6 mol% and DSPE_2K_/DTX-S-S-OA =11.4 mol%) was dissolved in 1 ml of anhydrous ethanol, then the solution was dropwise (1 ml/min) added to 4 ml of purified water under stirring (800 rpm) at room temperature. Consequently, the nanoassemblies were formed with light blue opalescence. Finally, ethanol was evaporated under reduced pressure at room temperature, then purified water was added to the concentrated suspension to obtain 4 mL of the final colloidal solution (1 mg/mL of prodrugs). Non-PEGylated prodrug NPs were prepared by the same method but without the addition of DSPE_2K_. The particle size and zeta potential of prodrug NPs before and after evaporation were measured in triplicate by a Malvern ZetaSizer (Nano ZS, Malvern, U.K). The results were represented as mean ± standard deviation (SD). The morphology of prodrug NPs was observed by using JEM-2100 trans-mission electron microscope (TEM, JEOL, Japan). 5 μl of NPs solution was dropped on a carbon-coated copper grid for 30 s and then dried with the aid of filter paper. Finally, the samples were stained with 1% phosphotungstic acid (5 μL) for 30 s and natural dried. The images of the samples were formed at 200 keV.

To demonstrate the influence of DSPE_2K_ to the colloidal stability of prodrug NPs, PEGylated prodrug NPs and non-PEGylated prodrug NPs were added to phosphate buffered saline (PBS) of pH 7.4and then the changes of the solution surface morphology were observed. To further evaluate the colloidal stability of prodrug NPs, 1 ml of DTX-OA/DSPE_2K_ NPs or DTX-S-S-OA/DSPE_2K_ NPs was added to 20 ml of PBS (pH 7.4) medium, containing 10% of fetal calf serum (FBS). The mixtures were incubated at 37 °C with mild shaking. At prescriptive intervals, the mean particle size was measured by Zetasizer. Besides, the long-term stability of prodrug NPs stored at 4 °C was investigated by observing the changes of average particle size.

### Self-assembly simulation

To evaluate the self-assembly mechanism, a computational simulation was used (Xue et al., 2016). The 2 D structures of the docetaxel-oleic acid conjugate (DTX-OA and DTX-S-S-OA) were created using Marvin sketch software (version: 16.4.25.0, Hungary), and the 3 D structures of molecules were built by optimizing the geometry minimization and the dynamic optimization with the Sybyl 6.9.1 software package (Tripos Associates: St. Louis, MO, 2003). The parameters optimized were: energy change 0.005 (kcal/mol) and max iterations 10,000 and assigned charges using the Gasteiger-Huckel method and minimized with the Powell method (Tripos force field) to an energy change of 0.005 kcal/(mol*Å) (Powell, 1977). All other parameters were maintained at the default values (Sun et al., 2016b; Zhang et al., 2017). The tetramers of DTX-OA and DTX-S-S-OA for molecular dynamics (MD) simulations were performed by Material Studio 8.0 software package. Firstly, the system containing 200 water molecules and four DTX conjugate molecules was established by employing amorphous cell module. The system was energy minimized using the forcite plus model. Finally, MD simulations were performed for 100 ns under the constant temperature of 298 K.

### In vitro *drug release*

The release of DTX from prodrug NPs was investigated at 37 °C with pH 7.4 PBS as release medium, containing 30% (v/v) ethanol with 0 μmol , 10 μmol or 10 mmol mmol dithiothreitol (DTT), respectively. Typically, DTX-OA/DSPE_2K_ NPs and DTX-S-S-OA/DSPE_2K_NPs were dispersed in 30 ml of the release medium with shaking (100 rpm). At the predetermined time points, 1 ml of sample solution was withdrawn and 10 μL of the samples were analyzed by HPLC (Hitachi HPLC system (Tokyo, Japan); mobile phase: acetonitrile: water (60:40, v/v); flow rate: 1 ml/min; chromatographic column: 150 × 4.6 mm, NUCLEOSIL 100-5 C18 (Macherey-Nagel, GmbH & Co. KG, Düren, Germany); wavelength: 230 nm; injection volume: 110 μL).

### Cell culture

4T1 breast cancer cells were cultured in Gibico 1640 medium with 10% FBS, penicillin (100 units/ml) and streptomycin (100 µg/ml), under a humidified atmosphere of 5% CO_2_. The cells were sub-cultured until 85% confluence by digestion of trypsin-EDTA and the culture medium was replaced once every two days.

### Cytotoxicity assay

The *in vitro* antitumor activity of DTX solutions, DTX-OA/DSPE_2K_ NPs and DTX-S-S-OA/DSPE_2K_ NPs was evaluated by MTT assays. Briefly, 4T1 cells (3 × 10^3^) were seeded in 96-well plates for 24 h. The next day, the complete medium was replaced by 200 µL of fresh medium with various concentrations of DTX or prodrugs and further incubated for 48 or 72 h, respectively. Then, the medium were replaced by adding 150 µL fresh culture medium and stained by 50 µL of MTT solution (5 mg/mL). After 4 h, 200 µL dimethylsulfoxide (DMSO) was added to replace the supernatant. The absorbance was detected at 490 nm by a microplate reader (Model 500, USA). The following equation was applied to calculate the inhibition rate and the IC_50_ was obtained by the SPSS software.

$$\text{Inhibition rate }\left(\%\right)=\left(\text{1}-{\text{A}}_{\text{sample}}/{\text{A}}_{\text{control}}\right)\times \text{1}00$$

### Cellular uptake and endocytosis mechanism

4T1 cells were seeded in 12-well plates at a density of 1 × 10^5^ cells/well. After incubation for 24 h, the cells were washed and followed by incubation with free C-6, C-6-labeled DTX-OA/DSPE_2K_ NPs and C-6-labeled DTX-S-S-OA/DSPE_2K_ NPs with an equivalent concentration of C-6 (250 ng/mL) at 37 °C for 0.5 h or 2 h. Afterward, the cells were rinsed thrice with cold DPBS. 4% formaldehyde was used to fix the cells. DAPI was used as the coloring matter for cell nucleus. After the samples being prepared, they were observed via confocal laser scanning microscopy (Zeiss LSM 510 Meta, Germany).

Similarly, 4T1 cells were pre-seeded in 12-well plates at a density of 1 × 10^5^ cells/well. After incubation for 24 h, the medium was replaced by free C-6, C-6-labeled DTX-OA/DSPE_2K_ NPs and C-6-labeled DTX-S-S-OA/DSPE_2K_ NPs with an equivalent concentration of C-6 ( 250 ng/ml) at 37 °C for 2 h. Then, the cells were washed with cold DPBS and analyzed by flow cytometer. To further explore the mechanism of cellular uptake, several specific endocytosis inhibitors were added to the pre-treating 4T1 cells for 1 h and subsequently incubated with C-6-labeled DTX-OA/DSPE_2K_ NPs and C-6-labeled DTX-S-S-OA/DSPE_2K_ NPs for 2 h. Finally, the results of intracellular fluorescence were measured by the FACSCalibur flow cytometer.

### Animal studies

All the animals were obtained from the Laboratory Animal Center of Shenyang Pharmaceutical University and all the animal experiments in this work were carried out according to the Guide for Care and Use of Laboratory Animals approved by the Institutional Animal Ethical Care Committee (IAEC) of Shenyang Pharmaceutical University.

### Pharmacokinetics and biodistribution

Sprague-Dawley (SD) rats (200–220 g) were used to carry out the pharmacokinetic studies. Thirty-six fasted rats were randomly divided into six groups. DTX solution, DTX-OA/DSPE_2K_ NPs and DTX-S-S-OA/DSPE_2K_ NPs at a single dose of 5 mg/kg with equivalent DTX were administrated intravenously. At predetermined predesigned time intervals, approximately 0.4 ml blood samples were transferred into heparin sodium tubes with heparin sodium from orbit and centrifuged at 13,000 rpm for 5 min to obtain plasma. The plasma samples obtained were stored at −20 °C until analysis. The concentration of free DTX and two prodrugs in rat plasma was determined by UPLC–MS/MS. BEH C18 column (50 mm ×2.1 mm, 1.7 μm) was employed for chromatographic separation. The mobile phase consisted of acetonitrile (A) and water (B) for chromatographic separation at a flow rate of 0.2 ml/min. To determine DTX, a gradient elution was performed: 0 ∼ 0.5 min, 30% A; 0.51 ∼ 3.0 min, 95% A; 3.1 ∼ 5 min, 30% A. To determine DTX-OA and DTX-S-S-OA, another gradient elution was performed: 0 ∼ 5 min, 90% A; 10% B. The injection volume was 5 μL and the temperature of column and autosampler was maintained at 40 and 4 °C, respectively.

To evaluate the tumor-targeting capability of prodrug NPs, 4T1 tumor bearing BALB/c mice were used and free DiR solution, DiR labeled DTX-OA/DSPE_2K_ NPs and DTX-S-S-OA/DSPE_2K_ NPs were intravenously administrated at a dose of 1.25 mg/kg equivalent to that of DiR. After post injection for 4 or 24 h the BALB/c mice were sacrificed and the major organs (heart, liver, spleen, lung and kidney) and tumors were harvested to carry out near-infrared fluorescence imaging by IVIS spectrum small-animal imaging system.

### In vivo *antitumor efficacy*

Female BALB/c mice (18–22 g) were used to carry out the *in vivo* antitumor experiment. 4T1 (5 x 10^6^) cells were subcutaneously injected into the right rear of mice to build the 4T1 tumor bearing mice models. When tumor volume reached approximately 100 mm^3^, the mice were randomly divided into four groups (*n* = 5) and were administered intravenously every two days with PBS (control), DTX solution, DTX-OA/DSPE_2K_ NPs and DTX-S-S-OA/DSPE_2K_ NPs at a dose equivalent to DTX of 6 mg/kg. Tumor volume and body weight were measured every day to evaluate the antitumor efficacy and systemic toxicity. After two days of the last treatment, the mice were killed and then the major organs and tumors were harvested and fixed by formalin to prepare tissue sections for hematoxylin and eosin (H&E). Tumors were weighed to calculate the tumor burden. Besides, the blood was taken and 0.2 ml serum was prepared to determine blood urea nitrogen (BUN), creatinine (CREA), aspartate transaminase (AST), alanine transaminase (ALT) and uric acid (UA).

### Data analysis

All the data in the research represented as mean ± standard deviation (SD). Statistical differences were performed through student’s *t*-test or one-way analysis of variance (ANOVA). *p < .05 represented statistically significant differences between two groups of data.

## Results and discussion

### Design and synthesis of DTX-S-S-OA

**Figure 1.**
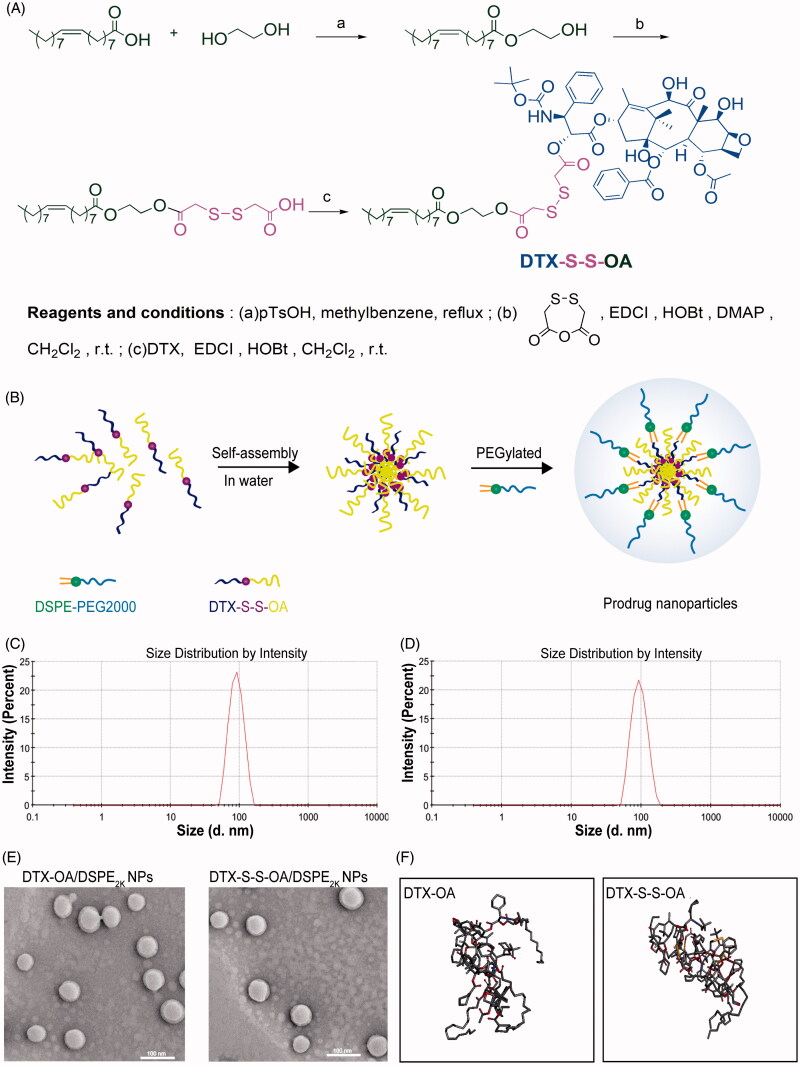
(A) Synthetic route of DTX-S-S-OA; (B) Preparation of prodrug NPs of DTX-S-S-OA; (C) Particle size of DTX-OA/DSPE2K NPs; (D) Particle size of DTX-S-S-OA/DSPE2K NPs; (E) Morphology of DTX-OA/DSPE2K NPs and DTX-S-S-OA/DSPE2K NPs by TEM; (F) MD simulations of tetramers of prodrugs.

DTX-S-S-OA was synthesized by conjugating DTX with oleic acid *via* a linkage of the disulfide bond (Figure 1(A)) and DTX-OA was developed by coupling DTX to oleic acid through a simple ester bond. MS and ^1^H NMR were used to confirm the chemical structures of DTX-S-S-OA and DTX-OA (Figure S(1,2)).

### Preparation and characterization of prodrug nanoassemblies

The nano-precipitation method was applied to prepare the prodrug NPs (DTX-OA NPs, DTX-S-S-OA NPs, DTX-OA/DSPE_2K_ NPs and DTX-S-S-OA/DSPE_2K_ NPs) (Figure 1(B); Ren et al., 2016a). The average size of these prepared NPs was 75 ∼ 95 nm (Figure 1 and Table S1) and the zeta potential of the prepared NPs was around −20 mV (Table S1) and the average size of these prepared NPs before and after evaporation did not change (Figure S3). The morphology of both non-PEGylated prodrug NPs and PEGylated prodrug NPs was performed by TEM and showed a spherical structure with the size of approximately 70 nm (Figure 1(E) and Figure S4). Compared to the traditional nano-formulations of DTX, DTX-OA/DSPE_2K_ NPs and DTX-S-S-OA/DSPE_2K,_ NPs possessed a higher drug loading of 60.2 and 50.4%, respectively (Table S1). The high drug loading efficacy would result in better therapeutic effect and lower excipient-associated toxicity.

To improve the stability of prodrug NPs and prolong the systemic circulation time in blood, DSPE_2K_ was added to the nano-formulation. The PEGylated prodrug NPs exhibited good stability in PBS (pH 7.4). On the contrary, the non-PEGylated prodrug NPs immediately separated crystals out when the prodrug NPs was added to PBS (pH 7.4) (Figure S5(A)). In addition, the PEGylated prodrug NPs, DTX-OA/DSPE_2K_ NPs and DTX-S-S-OA/DSPE_2K_ NPs, kept high stability in PBS (pH 7.4) containing 10% FBS at 37 °C for 12 h (Figure S5(B)). In the long-term stability study, the mean size of PEGylated prodrug NPs had no significant change when stored at 4 °C for 90 days (Figure S5(C)).

### Self-assembly simulation

Both DTX-OA and DTX-S-S-OA could self-assemble into NPs, but DTX would immediately precipitate in water without the aid of amphiphilic polymers. To explore the self-assembly mechanism of the oleate prodrug of DTX, molecular dynamics (MD) simulations for the tetramers of DTX-OA and DTX-S-S-OA were carried out (Wang et al., 2014). As shown in Figure 1(F), four single molecules quickly aggregated into a cluster of tetramer in water environment. As soon as the self-assembly was accomplished, the conformation remained unchanged all the time. All the generated movements were in the form of a cluster. It was apparently observed that the phenyl rings of DTX were curved inside the cluster and the unsaturated alkyl chains (OA) was rejected outside the cluster. On the basis of the structure of the tetramers, the noncovalent hydrophobic interactions and π–π stacking among DTX conjugates might be deemed to be the crucial factors that drove the self-assemble completely. The unsaturated alkyl chain of OA could provide flexible steric structures of DTX conjugates and balance the intermolecular forces (Figure S6). Therefore, when the self-assembly behavior of the DTX conjugates was performed, the adequate structural flexibility, hydrophobic interactions and potential intermolecular π–π stacking would promote the formation of a thermodynamic equilibrium state with the lowest possible energy state (Fischer et al., 2013).

### In vitro *drug release*

**Figure 2.**
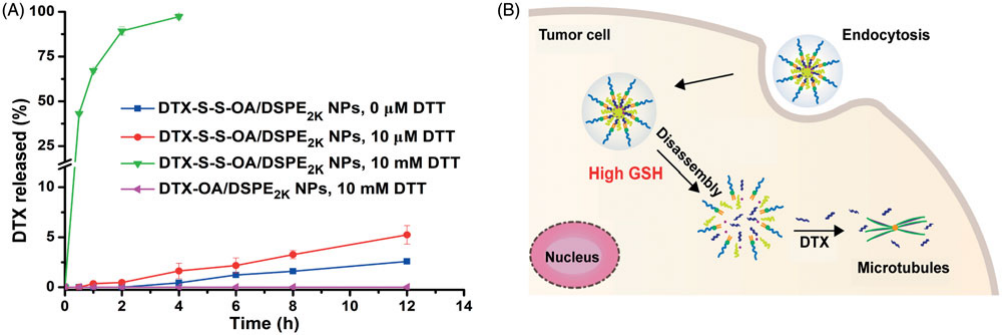
(A) Cumulative release of DTX from prodrug NPs in pH 7.4 with various concentrations of DTT (*n* = 3); (B) Schematic illustration of redox-responsive drug release of DTX-S-S-OA/DSPE2K NPs within tumor cells.

The *in vitro* redox-sensitive hydrolysis of DTX-S-S-OA/DSPE_2K_ NPs was investigated by using DTT. As shown in Figure 2(A), quite a little DTX was released from DTX-S-S-OA/DSPE_2K_ NPs without DTT and only about 5% DTX released from DTX-S-S-OA/DSPE_2K_ NPs in the existence of 10 µmol/L DTT in PBS (pH 7.4) for 12 h. However, almost all of the DTX was released from DTX-S-S-OA/DSPE_2K_ NPs with 10 mmol/L DTT within 4 h. In contrast, there was no DTX released from DTX-OA/DSPE_2K_ NPs in the presence of 10 mmol/L DTT within 12 h. The results revealed that DTX-S-S-OA/DSPE_2K_ NPs showed more rapid and selective release in redox environment (Figure 2(B)).

### Cytotoxicity assay

**Figure 3.**
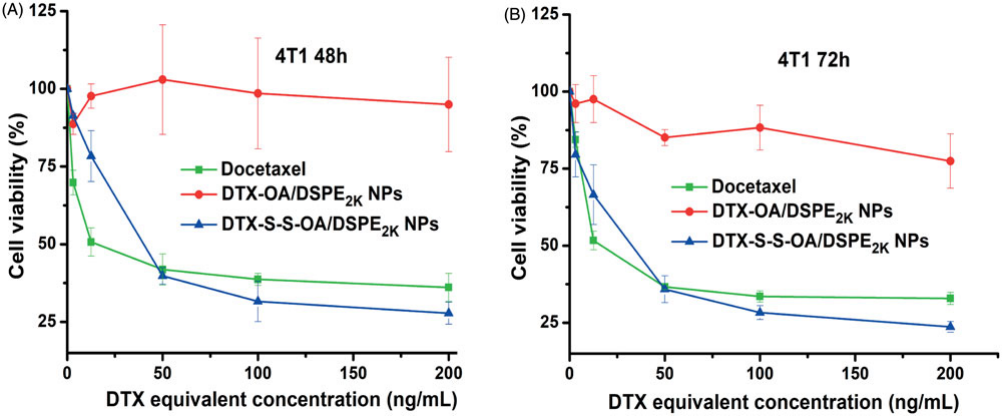
Cell viability treated with various concentrations of DTX solution, DTX-OA/DSPE2K NPs and DTX-S-S-OA/DSPE2K NPs after 48 h (A) or 72 h (B), respectively.

The *in vitro* cytotoxic activity of DTX solution, DTX-OA/DSPE_2K_ NPs and DTX-S-S-OA/DSPE_2K_ NPs was evaluated in 4T1 cells by MTT assay. As shown in Figure 3, there was no significant difference in terms of cytotoxicity against 4T1 cells between DTX solution and DTX-S-S-OA/DSPE_2K_ NPs. The comparable cytotoxicity might be related to the rapid drug release of DTX-S-S-OA/DSPE_2K_ NPs. By contrast, DTX-OA/DSPE_2K_ NPs demonstrated negligible cytotoxicity as the concentration increases due to its slow release rate of the drug. The more detailed half maximal inhibitory concentrations (IC_50_) were summarized in Table S2. The result demonstrated that the *in vitro* cytotoxic activity mainly depended on the drug release rate of DTX from the prodrug NPs. The concentration of GSH in tumor cells is higher than normal cells (Luo et al., 2016). Compared to DTX-OA/DSPE_2K_ NPs, DTX-S-S-OA/DSPE_2K_ NPs would perform a highly sensitive and selective release in tumor cells, facilitating significant growth inhibition of tumor cells (Figure 3).

### Cellular uptake and endocytosis mechanism

**Figure 4.**
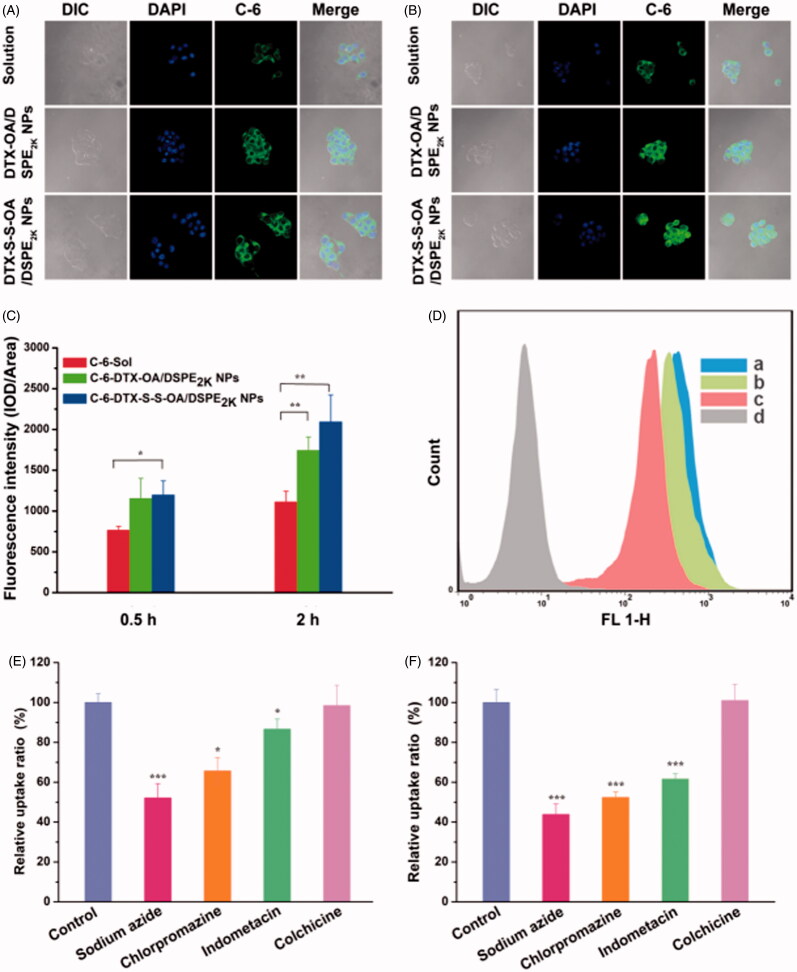
Confocal laser scanning microscopy (CLSM) images of 4T1 cells incubated with C-6solution or C-6labeled prodrug NPs for 0.5 h or 2 h, respectively (A,B); (C) Quantitative analysis for the fluorescent intensity of confocal laser scanning microscopy (*p < .05 and **p < .01, *n* = 3); (D) Flow cytometry results of cellular uptake of 4T1 cells after incubation for 2 h with (Blank control, C-6 solution, DTX-OA/DSPE2K NPs and DTX-S-S-OA/DSPE2K NPs); Cellular uptake results of pretreating 4T1 cells with several endocytosis inhibitors after incubation with DTX-OA/DSPE2K NPs (E) or DTX-S-S-OA/DSPE2K NPs (F) (*p < .05, **p < .01 and ***p < .001 vs. control group, *n* = 3).

To investigate the cellular uptake, C-6 solution, C-6-labeled DTX-OA/DSPE_2K_ NPs and C-6-labeled DTX-S-S-OA/DSPE_2K_ NPs were incubated with 4T1 cells for 0.5 or 2 h, respectively. As shown in Figure 4(A-D), the intracellular fluorescence intensity of prodrug NPs was higher to C-6 solution. Furthermore, both C-6 solution and C-6 labeled prodrug NPs showed the time-dependent cellular uptake on 4T1 cells.

Although prodrug-based NPs has been widely investigated and applied for efficient anticancer drug delivery, the detailed endocytosis mechanism of prodrug-nanoassemblies is still not clear. In this section, we studied the endocytosis mechanism of DTX-OA/DSPE_2K_ NPs and DTX-S-S-OA/DSPE_2K_ NPs on 4T1 cells by using several specific cellular uptake inhibitors. As shown in Figure 4(E,F), sodium azide drastically suppressed the internalization of DTX-OA/DSPE_2K_ NPs and DTX-S-S-OA/DSPE_2K_ NPs in 4T1 cells, suggesting that the endocytosis of the prodrug NPs was an energy-dependent process (Matsubara et al., 2017). Besides, chlorpromazine and indometacin could also significantly reduce the cellular uptake of the prodrug NPs, suggesting that both clathrin-mediated and caveolae-mediated endocytosis contributed to the cellular uptake of prodrug NPs. Colchicine almost did not affect the cellular uptake process of prodrug NPs. Therefore, multiple endocytosis mechanisms were involved in the cellular uptake of prodrug-nanoassemblies with energy-dependence.

### Pharmacokinetics and biodistribution

**Figure 5.**
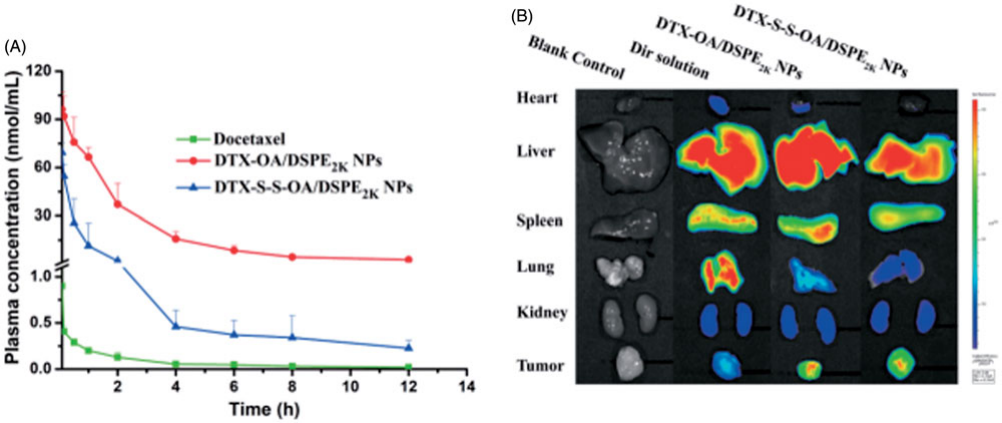
(A) Plasma concentration-time profiles of DTX solution, prodrug nanoassemblies after a single intravenous administration of 5 mg/kg (DTX equivalent) (*n* = 6); (B) *In vivo* fluorescent distribution images of DiR solution and DiR-labeled prodrug nanoassemblies at 24 h.

The pharmacokinetics study of DTX and prodrug NPs were determined using the UPLC-MS/MS method. The plasma drug concentration-time curves (AUC) of the compounds were shown in Figure 5(A) and S7 and the primary pharmacokinetic parameters were presented in Table S3. As shown in Figure 5(A), the Taxotere (DTX solution) showed short retention time in plasma and was rapidly eliminated. On the contrary, DTX-OA/DSPE_2K_ NPs and DTX-S-S-OA/DSPE_2K_ NPs significantly prolonged the systemic circulation in the blood (Mishra et al., 2016). Compared to Taxotere, PEGylated prodrug NPs had higher AUC and longer retention time in the systemic circulation (Table S3).

Prolonged circulation time in blood of prodrug NPs would facilitate drug accumulation in tumor *via* EPR effect. Therefore, the BALB/c mice bearing 4T1 tumor were utilized to further study the *ex vivo* biodistribution of DiR-labeled prodrug NPs. The fluorescence of organs and tumors was evaluated using noninvasive live animal imaging technology. As shown in Figure 5(B) and S8, there was no significant difference in tumor accumulation among different formulations at 4 h, but 24 h after administration, free DiR solution showed high accumulation in lung, whereas very weak fluorescent intensity was observed in tumor region. By comparison, the strong fluorescence signal was observed in tumor site with DiR labeled DTX-OA/DSPE_2K_ NPs and DiR labeled DTX-S-S-OA/DSPE_2K_ NPs. These results demonstrated that extended circulation time could facilitate tumor accumulation of prodrug-nanoassemblies *via* EPR effect.

### In vivo *antitumor efficacy*

**Figure 6.**
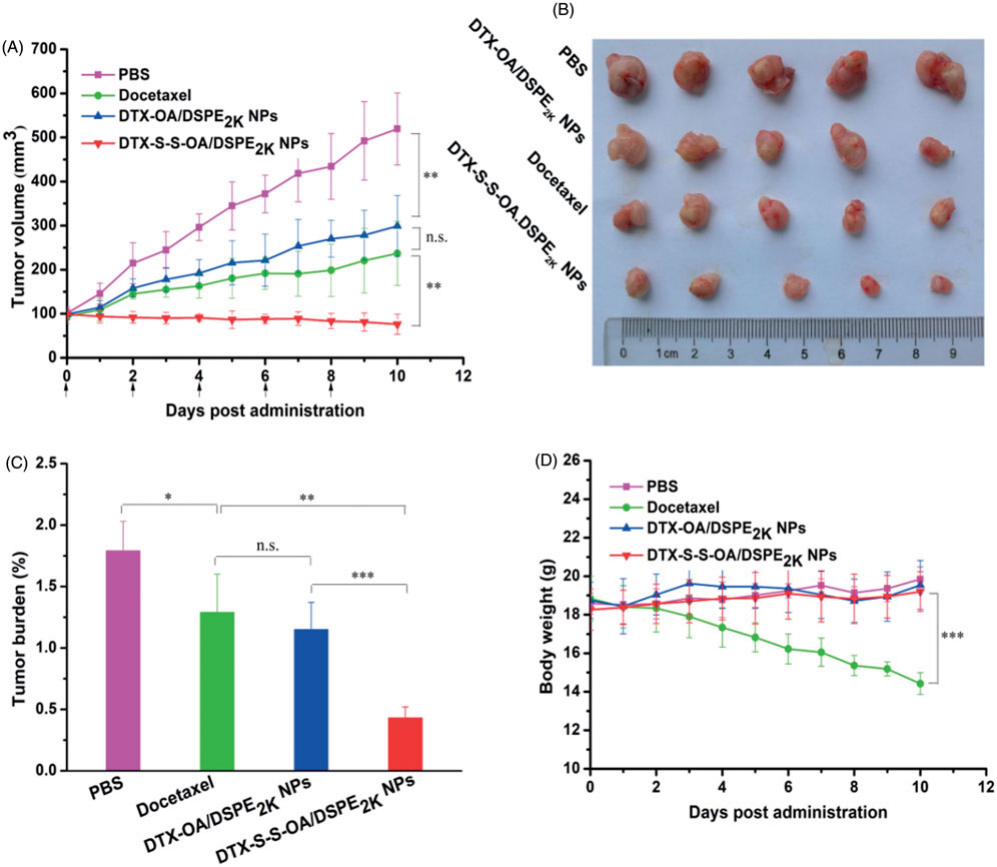
Evaluation on the *in vivo* antitumor activity against 4T1 xenograft tumors. (A) The changes of the tumor volume after various treatments; (B) Images of tumors after the last treatment; (C) Tumor burden after the last treatment, tumor burden = tumor weight/average body weight; (D) Body weight variations during treatment (*p < .05, **p < .01 and ***p < .001, *n* = 5).

The *in vivo* antitumor efficacy of the prodrug NPs was further investigated using a xenograft model of 4T1 breast cancer in the BALB/c mice. As shown in Figure 6(A–C), the mice treated with DTX-S-S-OA/DSPE_2K_ NPs showed significant suppression on tumor growth (∼80 mm^3^ on day 10), suggesting that DTX-S-S-OA/DSPE_2K_ NPs performed potent antitumor activity against 4T1 breast cancer. The good antitumor efficacy of DTX-S-S-OA/DSPE_2K_ NPs should be attributed to multiple therapeutic advantages of this prodrug-nanosystem, including high drug-loading, prolonged circulation time in blood, high-efficient cellular uptake and redox-sensitive drug release. In contrast, DTX-OA/DSPE_2K_ NPs and DTX solution only moderately delayed the tumor growth when compared to the control group (PBS), the tumor volume reached about 300 and 250 mm^3^ on day 10, respectively. The slow drug release from DTX-OA/DSPE_2K_ NPs and rapid clearance of DTX solution might result in inferior therapeutic effect of DTX-OA/DSPE_2K_ NPs and DTX solution. An obvious weight loss treated with DTX solution was shown in Figure 6(D), but no significant change in body weight was observed in the group of prodrug NPs.

As shown in Figure S7, H&E-stained pathological section results showed that several metastatic lesions were observed in the liver of the mice treated with PBS and DTX-OA/DSPE_2K_ NPs, but no metastatic lesion was found in the liver of the mice treated with DTX solution and DTX-S-S-OA/DSPE_2K_ NPs. These results suggested that DTX-S-S-OA/DSPE_2K_ NPs not only showed potent antitumor activity against the primary breast tumor but also could efficiently inhibit metastasis of breast cancer. Moreover, as shown in Figure S9, different levels of apoptosis and necrosis were found in the tumor sections of DTX solution, DTX-OA/DSPE_2K_ NPs and DTX-S-S-OA/DSPE_2K_ NPs groups. As shown in Figure S10, no noticeable change in hepatic and renal function was observed from the hematological parameters of all the mice. Therefore, DTX-S-S-OA/DSPE_2K_ NPs demonstrated higher antitumor activity against 4T1 breast cancer with lower toxicity when compared with both DTX solution and DTX-OA/DSPE_2K_ NPs.

## Conclusion

In the present study, a novel disulfide bond bridged oleate prodrug of DTX (DTX-S-S-OA) was designed and synthesized to construct prodrug-nanoassemblies for breast cancer therapy. The self-delivering prodrug-based nanosystem (DTX-S-S-OA/DSPE_2K_ NPs) demonstrated multiple drug delivery advantages, including high drug loading capacity, redox-responsive drug release, increased cellular uptake and comparable cytotoxicity in 4T1 breast cancer cells when compared with free DTX. The endocytosis mechanism of prodrug-nanoassemblies was investigated in the present study for the first time. It turned out that multiple endocytosis mechanisms involved in the cellular uptake of prodrug-nanoassemblies with energy-dependence which included clathrin-mediated and caveolae-mediated endocytosis. *In vivo*, DTX-S-S-OA/DSPE_2K_ NPs demonstrated significantly prolonged systemic circulation, increased accumulation in tumor site, resulting in a potent antitumor activity in 4T1 breast cancer xenograft in BALB/c mice. Therefore, prodrug NPs self-assembled from disulfide bond bridged oleate prodrug of DTX showed potent antitumor activity against its major indication (breast cancer) *in vitro* and in *vivo*, and provided great potential for clinical breast cancer therapy.

## Supplementary Material

IDRD_Sun_et_al_Supplemental_Content.doc
