# Supplementary material for: Self-delivering prodrug-nanoassemblies fabricated by disulfide bond bridged oleate prodrug of docetaxel for breast cancer therapy
Source: Drug Deliv. 2017 Sep 26;24(1):1460–9. doi: 10.1080/10717544.2017.1381201 (PMC8241025; doi:10.1080/10717544.2017.1381201)
Supplement: IDRD_Sun_et_al_Supplemental_Content.doc [file IDRD_A_1381201_SM5083.doc]

**Supporting Information**

**Self-delivering prodrug-nanoassemblies fabricated by disulfide bond bridged oleate prodrug of docetaxel for breast cancer therapy**

Authors: Shenwu Zhang^1^, Jibin Guan^1^, Mengchi Sun^1^, Dong Zhang^1^, Haotian Zhang^3^, Bingjun Sun^1^, Weiling Guo^1^, Bin Lin^2^, Yongjun Wang^1^, Zhonggui He^1^, Cong Luo^*,1^, Jin Sun^*,1^

Affiliations:

^1^Department of Pharmaceutics, Wuya College of Innovation, Shenyang Pharmaceutical University, Shenyang, Liaoning, 110016, P. R. China

^2^Key Laboratory of Structure-Based Drug Design and Discovery, Shenyang Pharmaceutical University, Ministry of Education, Wenhua Road, No. 103, Shenyang, 110016, China

^3^School of Life Science and Biopharmaceutics, Shenyang Pharmaceutical University, Wenhua Road, No. 103, Shenyang 110016, China

*Corresponding authors:

Prof. Jin Sun, Ph.D. and Dr. Cong Luo, Ph.D.

Wuya College of Innovation, Shenyang Pharmaceutical University, Shenyang, Liaoning, 110016, P. R. China

Tel: +86-24-23986321; Fax: +86-24-23986321

E-mail address: [sunjin66@21cn.com](mailto:sunjin66@21cn.com); [luocong_pharm@aliyun.com](mailto:luocong_pharm@aliyun.com)


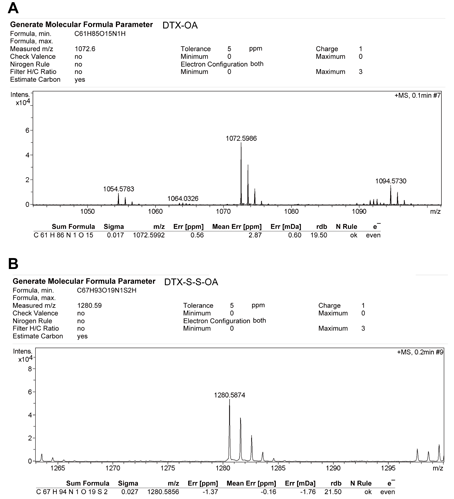


**Fig. S1** TOF-MS spectra of DTX-OA and DTX-S-S-OA


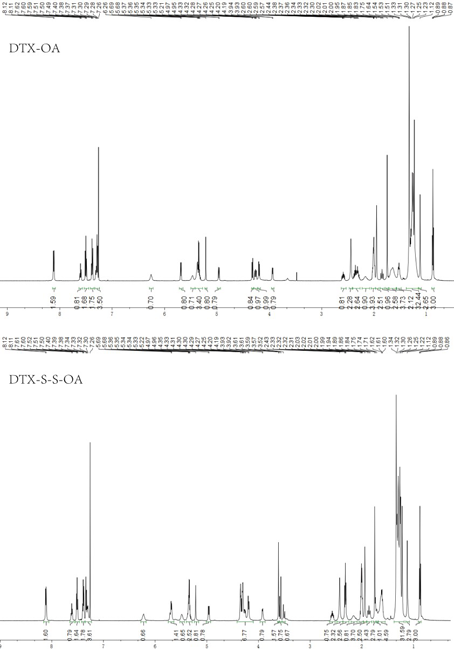


**Fig. S2** ^1^H NMR spectra (600 MHz, CDCl_3_) of DTX-OA and DTX-S-S-OA.

**^1^H NMR results:**

The ^1^H NMR spectra of DTX-OA showed clearly characteristic peaks of OA: δ 0.88 (t, 3H, -CH_3_), δ 1.39 – 1.16 (m, 10H, CH_3_-(CH_2_)_5_-CH_2_), δ 1.39 – 1.16 (m, 8H, CH_3_-(CH_2_)_5_-CH_2_, -CH_2_-(CH_2_)_3_-CH_2_-), δ 2.06 – 1.98 (m, 4H, -CH_2_CH=CHCH_2_-), δ 5.35 (m, 2H, -CH=CH-) and DTX: 1.39 – 1.16 (m, 9H, -C(CH_3_)_3_ ), δ 3.94 (d, 1H, -H_3_)，δ 4.96 (d, 1H, -H_5_), δ 6.26 – 7.6 (m, 10H, 2C_6_H_6_), δ 8.11 (d, 2H, -Ar-H_25,29_), demonstrating the OA was successfully conjugated with DTX.

The ^1^H NMR spectra of DTX-S-S-OA showed distinctly characteristic peaks of OA: δ 0.88 (t, 3H, -CH_3_), δ 1.38 – 1.18 (m, 10H, CH_3_-(CH_2_)_5_-CH_2_), δ 1.38 – 1.18 (m, 8H, CH_3_-(CH_2_)_5_-CH_2_, -CH_2_-(CH_2_)_3_-CH_2_-), δ 2.06 – 1.98 (m, 4H, -CH_2_CH=CHCH_2_-), δ 5.35 (m, 2H, -CH=CH-), DTX: 1.39 – 1.16 (m, 9H, -C(CH_3_)_3_ ), δ 3.94 (d, 1H, -H_3_), δ 4.96 (d, 1H, -H_5_), δ 6.26 – 7.6 (m, 10H, 2C_6_H_6_), δ 8.11 (d, 2H, -Ar-H_25,29_) and the linkage: δ 3.51 – 3.92 (m,4H,-CH_2_S_2_CH_2_-), δ 4.10 – 4.42 (m, 4H, -OCH_2_CH_2_O-), demonstrating DTX-S-S-OA was successfully synthesized.


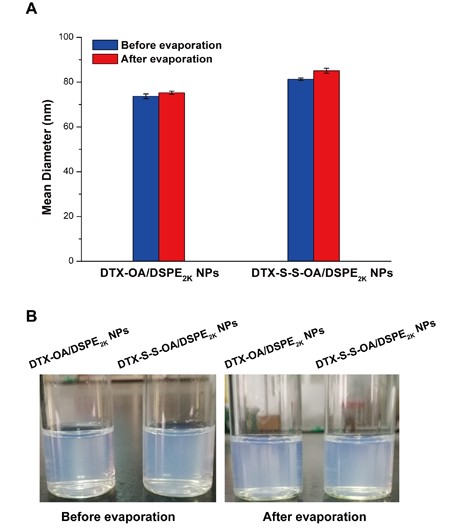


**Fig. S3** (A) Particle size of PEGylated prodrug NPs before and after evaporation; (B) Appearance of PEGylated prodrug NPs before and after evaporation.


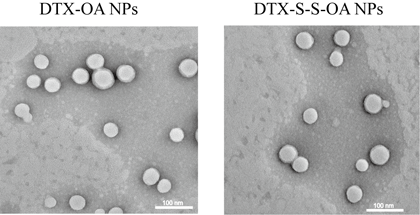


**Fig. S4** Morphology of non-PEGylated prodrug NPs (DTX-OA NPs and DTX-S-S-OA NPs) by TEM.


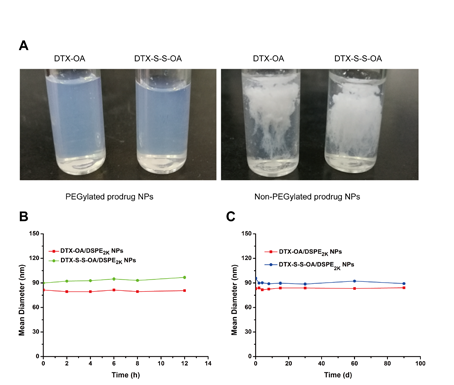


**Fig. S5** (A) Colloidal stability of PEGylated prodrug NPs and non-PEGylated prodrug NPs in PBS (pH 7.4); (B) Stability of prodrug NPs after incubation in pH 7.4 PBS containing 10% FBS at 37 °C for 12 h; (C) Long-term stability of prodrug NPs after store at 4 °C for 90 days.


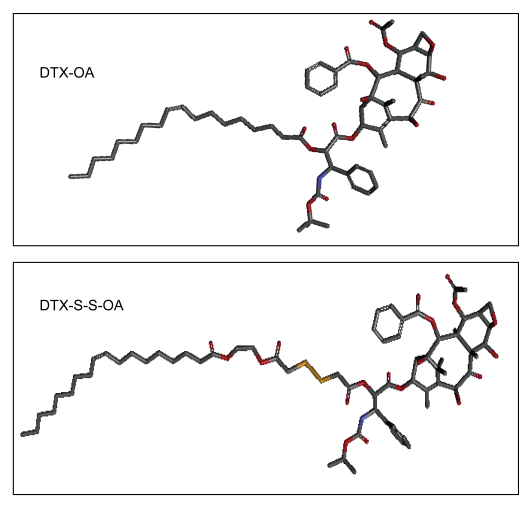


**Fig. S6** 3D chemical structures of DTX-OA and DTX-S-S-OA.


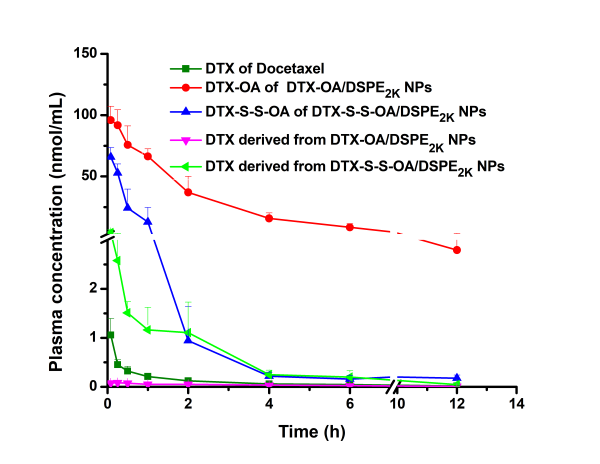


**Fig. S7** *In vivo* plasma concentration-time profiles of DTX, DTX-OA and DTX-S-S-OA following a single [tail](../../../../D:/Users/zhang/AppData/Local/Youdao/Dict/7.1.0.0421/resultui/dict/%3Fkeyword=tail) [intravenous](../../../../D:/Users/zhang/AppData/Local/Youdao/Dict/7.1.0.0421/resultui/dict/%3Fkeyword=intravenous) [injection](../../../../D:/Users/zhang/AppData/Local/Youdao/Dict/7.1.0.0421/resultui/dict/%3Fkeyword=injection) with Docetaxel solution, DTX-OA/DSPE_2K_ NPs or DTX--S-S-OA/DSPE_2K_ NPs at a DTX equivalent dose of 5mg/kg (n=6).


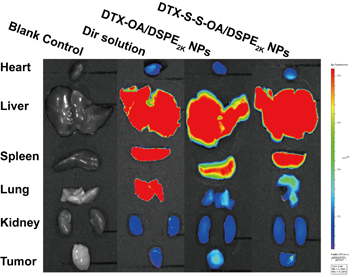


**Fig. S8** *In vivo* fluorescent distribution images of DiR solution and DiR-labeled prodrug nanoassemblies at 4 h.


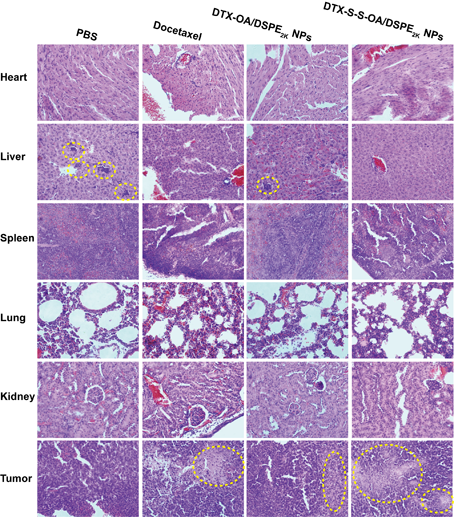


**Fig. S9** The images of different organ sections and tumors stained with H&E after various treatments.


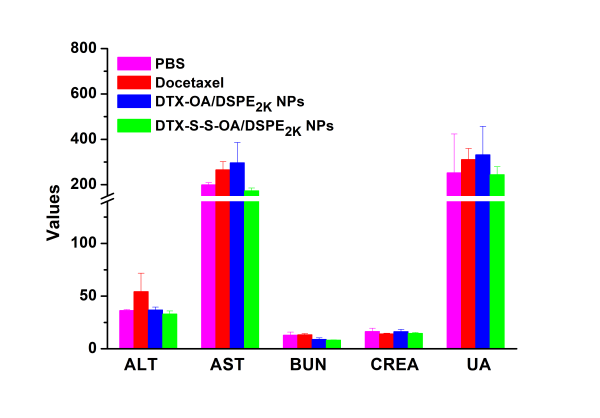


**Fig. S10** Liver and kidney functional parameters after the last treatment.

**Table S1** Characterization of non-PEGylated prodrug NPs and PEGylated prodrug NPs.

| Formulations | Size (nm) | PDI | Zeta potential (mV) | DL (%) |
| --- | --- | --- | --- | --- |
| DTX-OA NPs | 75.70±0.4 | 0.09±0.1 | -17.8±0.3 | 75.3 |
| DTX-S-S-OA NPs | 81.76±1.0 | 0.12±0.0 | -14.13±1.6 | 63.0 |
| DTX-OA/DSPE_2k_ NPs | 81.67±0.5 | 0.14±0.0 | -18.27±1.6 | 60.2 |
| DTX-S-S-OA/DSPE_2k_ NPs | 85.10±1.1 | 0.14±0.0 | -20.43±1.2 | 50.4 |

**Table S2** IC_50_ of DTX solution, DTX-OA/DSPE_2K_ NPs and DTX-S-S-OA/DSPE_2K_ NPs against 4T1 cells at 48 or 72 h (n = 3).

| Formulations | 4T1 | |
| --- | --- | --- |
|  | 48h | 72h |
| Docetaxel | 25.1±7.1 | 23.5±4.9 |
| DTX-OA/DSPE_2K_ NPs | >200 | >200 |
| DTX-S-S-OA/DSPE_2K_ NPs | 45.6±14.9 | 32.2±13.3 |

**Table S3** Pharmacokinetic parameters of DTX and prodrug-based nano-formulations (n = 6).

| Formulations | Determined  drug | C_max_  (nM/ml) | T_max_(h) | AUC_0-24_ (nM· h /ml) | T_1/2_ (h) |
| --- | --- | --- | --- | --- | --- |
| Docetaxel | DTX | 0.41±0.1 | 0.25±0.0 | 0.94±0.2 | 3.2±1.1 |
| DTX-OA/DSPE_2k_ NPs | DTX | 0.09±0.0 | 0.29±0.1 | 0.40±0.1 | 4.33±1.4 |
|  | DTX-OA | 93.08±13.1 | 0.29±0.1 | 236.58±24.5 | 3.03±0.9 |
| DTX-S-S-OA/DSPE_2k_ NPs | DTX | 2.58±0.8 | 0.25±0.0 | 5.54±1.3 | 2.69±1.3 |
|  | DTX-S-S-OA | 52.02±7.3 | 0.25±0.0 | 49.19±13.6 | 5.73±6.0 |
